# Exploring potential antidiabetic and anti-inflammatory flavonoids from *Euphorbia humifusa* with an integrated strategy

**DOI:** 10.3389/fphar.2022.980945

**Published:** 2022-08-29

**Authors:** Tojofaniry Fabien Rakotondrabe, Minxia Fan, Mingquan Guo

**Affiliations:** ^1^ CAS Key Laboratory of Plant Germplasm Enhancement and Specialty Agriculture, Wuhan Botanical Garden, Chinese Academy of Sciences, Wuhan, China; ^2^ University of Chinese Academy of Sciences, Beijing, China; ^3^ Sino-Africa Joint Research Center, Chinese Academy of Sciences, Wuhan, China; ^4^ Innovation Academy for Drug Discovery and Development, Chinese Academy of Sciences, Shanghai, China

**Keywords:** *Euphorbia humifusa*, antidiabetic, anti-inflammatory, bio-affinity, flavonoid, ligand

## Abstract

*E. humifusa* Willd, a monoecious annual plant, native to Eastern Asia, has been traditionally attributed to the treatment and prevention of miscellaneous diseases, including diabetes mellitus and its associated complications. Earlier studies have supported this species’ pharmacological efficacies including its antibacterial, antidiabetic, and anti-inflammatory properties. Even so, the underlying bioactive components with their mechanisms of action associated with its antidiabetic and anti-inflammatory effects remain elusive. The preamble *in vitro* assessments of the crude extract and its different fractions revealed that the *n*-butanol fraction (EHNB) exhibited the best activity, which was subsequently subjected to a rapid screening of candidate ligands through bio-affinity ultrafiltration with the two enzyme targets: α-glucosidase (α-Glu) and cycloxygenase-2 (COX-2) combined with UPLC/QTOF-MS. As a result, 7 compounds were identified from EHNB, among them, vitexin and astragalin were screened out as the most active ligand compounds. Vitexin showed great specific binding (SB) affinity values of 1.26 toward α-Glu and 1.32 toward COX-2, while astragalin showed 1.32 and 1.36, respectively. The docking simulation results exhibited strong interactions of vitexin and astragalin with the key residues of the enzyme targets, suggesting their possible mechanisms of action. The *in vitro* antidiabetic validation revealed noticeable half-maximal inhibitory effects (IC_50_) of 36.38 ± 3.06 µM for vitexin and 42.47 ± 4.13 µM for astragalin, much better than that of the positive drug acarbose (109.54 ± 14.23 µM). Similarly, these two compounds showed the inhibitory activity against COX-2 with the half-maximal inhibitory effects (IC_50_) at 27.91 ± 1.74 µM and 49.05 ± 1.49 µM, respectively. Therefore, these two flavonoid compounds (vitexin and astragalin) were speculated as potential antidiabetic and anti-inflammatory compounds from *E. humifusa*. Taken together, the integrated strategy applied to *E. humifusa* led to the fast identification of two potential double-acting flavonoids and enlightened its antidiabetic and anti-inflammatory uses. Besides these findings, the integrated strategy in this study could also be used to facilitate the rapid discovery and development of active candidates from other traditional herbal medicines against multi-drug targets and to aid in revealing their mechanisms of action for their traditional uses.

## 1 Introduction

Diabetes mellitus (DM) is a chronic metabolic disease characterized by reduced insulin sensitivity or insulin deficiency, causing blood hyperglycemia in the postprandial and fasting state (Kazeem et al., 2021). By 2045, it is projected that around 800 million of the global population will be hurt by this pandemic metabolic disorder with 6.7 million recorded deaths in 2021 (Li et al., 2017; Ni et al., 2022). Persistent hyperglycemia encompasses multiple complications leading to nephropathy, retinopathy, neuropathy and vascular damage (Yen et al., 2021). Inflammation, oxidative stress, and obesity are among the related symptoms that are crucial in the pathophysiology of DM (Ma et al., 2018; Paun et al., 2020; Zhang et al., 2021). Therefore, researchers thrive on the quest and development of safe and cost-effective drugs to address DM issues and especially to limit its related complications.

From ancient times, herbal medicines have played significant roles in managing diverse health disorders including DM. The available pharmacotherapy enables the treatment of DM from different approaches, but in many cases there has been an increase of side effects such as gastrointestinal discomfort, hypoglycemia, and dizziness. These factors encourage researchers to seek new and safer alternatives from natural resources. Recent discovery evolving the uses of the powerful screening tool, based on bio-affinity ultrafiltration, has helped researchers to bypass the labor-intensive and time-consuming drug discovery process (Ye et al., 2022). This innovative approach consists of promoting the ligand-macromolecule complex formation between a mixture and a drug target, then fishing out the ligands candidates *via* centrifugal ultrafiltration, followed by their identification on analytical instruments like UPLC/QTOF-MS (Feng et al., 2021).

*E. humifusa* Willd, commonly called creeping euphorbia, is a monoecious annual plant native to eastern Asia (Kim et al., 2016). Growing up to 20 cm in height and generally glabrous, the plant presents a fine slender multiple branched stems and opposite leaves (Pahlevani and Riina, 2011). According to the Chinese pharmacopeia (2020 edition), this species has been attributed to the treatment of multifarious ailments such as dysentery, enteritis, and traumatic bleeding. In addition, previous research has supported the pharmacological potencies of this plant including its antibacterial, antidiabetic, and anti-inflammatory activities (Kang et al., 2012; Tian et al., 2019). However, this species bioactive components together with their associated mechanisms of action in terms of its antidiabetic and anti-inflammatory uses need to be explored. Hence, the purpose of this present study was to rapidly investigate the potential bioactive components of *E. humifusa* and elicit their possible modes of action. A preamble *in vitro* assessment of different *E. humifusa* extracts led us to depict the powerful active *n*-butanol fraction (EHNB) which was subsequently subjected to bio-affinity ultrafiltration combined with UPLC/QTOF-MS. Thereafter, docking simulations were used to investigate the interactions between the compounds with high affinity and the active pocket of the target enzymes α-Glu and COX-2. The lead compounds vitexin and astragalin’s capacities to mitigate the two target enzymes functions were confirmed *in vitro* with noticeable IC_50_ values as compared to the positive drugs. Despite the attempt to generate scientific support for the empirical virtues of *E humifusa*, this work offers also a valuable strategy for discovery and development of bioactive components from traditional herbal medicinal herbs.

## 2 Materials and methods

### 2.1 Plant materials preparation and extraction

Whole plant materials of *E. humifusa* were harvested around Wuhan Botanical Garden (Wuhan, China) in September 2020. A herbarium voucher specimen (No: 20200923) was prepared and authenticated by a senior taxonomist, Professor Guangwan Hu of the Key Laboratory of Plant Germplasm Enhancement and Agriculture Specialty (Wuhan Botanical Garden, Chinese Academy of Sciences). The samples were cleansed, shade-dried, and milled into powder before storage in an airtight recipient.

The extraction procedure of *E. humifusa* powder (400 g) was performed through an ultrasonic bath with 70% ethanol (ratio 1:8, w:v) for three consecutive times. The combined filtrates were evaporated under low pressure and lyophilized to afford the crude extract (EHCE: 93.22 g). Thereafter, from 20 g of EHCE were conducted sequential liquid-liquid separation yielding to petroleum ether fraction (EHPE: 2.20 g), ethyl acetate fraction (EHEA: 4.40 g), *n*-butanol fraction (EHNB: 3.74 g), and water fraction (EHWA: 5.02 g) after evaporation.

### 2.2 Chemicals and reagents

The extraction solvents ethanol, petroleum ether, ethyl acetate *n*-butanol and the dimethyl sulfoxide (DMSO) were supplied by Shanghai Chemical Reagent Corp. (Shanghai, China). The different standards used for the assays comprising gallic acid, rutin, ascorbic acid (Vit C), butylated hydroxytoluene (BHT), Trolox, 2,2-diphenyl-1-picrylhdrazyl (DPPH), 2,2′-azinobis-(3-ethyl-benzthiazoline-6-sulfonic acid) diammonium salt (ABTS), 2,4,6-Tri (2-pyridyl)-1,3,5-triazine (TPTZ), and indomethacin with a purity ≥99.5% were purchased from Sigma-Aldrich Corp (Shanghai, China). The HPLC grade solvents acetonitrile (ACN), methanol, and formic acid (FA) were obtained from TEDIA Company Inc. (Fairfield, OH, United States). The *p*-nitrophenyl α-D-glucopyranoside (*p*-NPG) substrate was acquired from Aladdin Bio-Chem Technology Corp. The COX-2 inhibitor screening assay kit (No: S0168) was purchased from Beyotime Biotechnology (Shanghai, China). The α-glucosidase enzyme was bought from Sigma Aldrich (St Louis, MO, United States) whereas Wuhan Antgene Biotechnology Co., Ltd. (Wuhan, China) supplied the COX-2 enzyme. The centrifugal ultrafiltration membranes 10 kDa Cut-off (YM-10) were purchased from Millipore Co. Ltd. (Bedford, MA, United States). Finally, Shanghai Macklin Biochemical Co. (Shanghai, China) supplied the phytochemical standards vitexin and astragalin (≥98%).

### 2.3 Total phenolic content and total flavonoid content

The phenolic contents of *E. humifusa* extract and its fractions were evaluated by Folin-Ciocalteu method, slightly modified from a previous report by Fan and coworkers (Fan et al., 2020). In brief, the sample was incubated with Folin-Ciocalteu reagent (10%, v/v) and sodium carbonate (7.5%, w/v) before 60 min incubation in the dark. The absorbance of the mixture was then read on a UV spectrophotometer set at a wavelength of 760 nm (UV 1100, MAPADA Shanghai, China). Gallic acid was used as the calibration standard, and the results were presented as its equivalent per gram of dried sample (mg GAE/g). The Aluminium chelating colorimetric method was performed for the estimation of total flavonoid content using rutin for the standard curve (Xu et al., 2019). An equal volume of the sample and sodium nitrite were mixed with 10% (w/v) AlCl_3_.6H_2_O. Thereafter, the mixture was supplemented with sodium carbonate (4%, w/v) and then incubated in the dark for 15 min. The absorbance of the mixture was finally read on a UV spectrophotometer set at a wavelength of 510 nm. The obtained results were denoted by milligram rutin equivalent per gram of dried sample (mg RE/g).

### 2.4 Antioxidant activity assay

The antioxidant activity of our samples was ascertained by evaluating their capacity to scavenge free radicals and reduce ferric ions. The DPPH and ABTS free radical scavenging assays were performed as per the method of Xu and his colleagues (Xu et al., 2019). Vit C, Trolox, and BHT were utilized as positive controls whereas pure methanol was for the blank control. The scavenging rate was calculated using the equation:

$$\;\text{Scavenging}\;\text{rate}\;\left(\%\right)\;=\frac{\left({\text{A}}_{0}\;-{\text{A}}_{S}\right)}{{\text{A}}_{0}}\times \;100\%$$

With A_*0*_ and A*s* are ascribed to the absorbance value of the blank control, and the tested sample or positive control respectively. The results were expressed in IC_50_ values (*n* = 3, mean ± standard deviation).

The ferric chelating behavior using the ferric reducing antioxidant power (FRAP) assay was conducted based on a priorly reported method by our research group (Rakotondrabe et al., 2022). The calibration curve was plotted from a range dilution of FeSO_4_·7H_2_O (0.06–1.50 mM) standard and the results were expressed in Millimolar Fe^2+^ per gram (mM Fe^2+/^g) of three replicate assays.

### 2.5 *In Vitro* α-Glu inhibition assay

The α-Glu inhibition activity of *E. humifusa* was determined through a minor modified method from a previous report (Liu et al., 2020). In brief, 150 µL phosphate buffer (pH 6.8) was mixed in a 96 well plate transparent with 25 µL of α-Glu enzyme solution (0.26 units/mL) and 25 µL of the diluted sample (DMSO: 5%, v/v). Then, the mixture was incubated for 15 min at 37°C before supplementation with 50 µL *p*-NPG (0.3125 mM) substrate to initiate the reaction. An incubation for another 15 min was monitored prior to the reaction termination with an addition of 50 µL sodium carbonate 200 mM. In the end, the absorbance was immediately read at 405 nm in a microplate reader (Tecan infinite M200 Pro). The α-Glu inhibition rate was figured using the following equation:

$$\text{Inhibition}\;\text{rate}\;\left(\%\right)\;=\;100\;-\left[\frac{\left({\text{A}}_{S}-{\text{A}}_{Sc}\right)}{\left({\text{A}}_{C}-{\text{A}}_{Bc}\right)}\times 100\right]\;\;\;\;$$

With A_*S*_ and A_*Sc*_ representing the absorbance of the sample test and the sample control while A_*C*_ and A_*Bc*_ for the absorbance of the control and the blank control accordingly.

### 2.6 *In Vitro* COX-2 inhibition assay

The *E. humifusa* anti-inflammatory activity was ascertained by a COX-2 inhibitor screening kit (Beyotime S0168). In brief, the working solutions including COX-2 cofactor, COX-2 enzyme solution, COX-2 substrate, and COX-2 probe were first prepared in accordance with the supplier manual, which were subsequently diluted ten times with the buffer kit assay. Thereafter, 150 µL Tris-HCl (pH 7.8), 10 µL of COX-2 cofactor, and 10 µL of work solution excluding the blank group were mixed in a 96 well black plate. The mixture was supplemented with 10 µL of samples or positive control (indomethacin) and instead, DMSO was used for the blank control and the 100% enzyme groups and mixed vigorously. After a 10 min incubation at 37°C, the reaction was initiated with 10 µL of COX-2 probe and 10 µL of COX-2 substrate. Finally, their relative fluorescence unit (RFU) was read from an excitation wavelength of 560 nm and an emission wavelength of 590 nm, after 20 min of incubation (37°C). The COX-2 inhibition rate was calculated from the equation:

$$\text{COX}-2\;\text{inhibition}\;\text{rate}\;\left(\%\right)\;=\frac{\left(\text{RF}{\text{U}}_{100\%\;enzyme}\;-\;\text{RF}{\text{U}}_{sample}\right)}{\left(\text{RF}{\text{U}}_{100\%\;enzyme}\;-\;\text{RF}{\text{U}}_{blank\;control}\right)}\;\times \;100\%\;\;\;\;$$

With RFU_*100% enzyme*_, RFU_*sample*_, and RFU_*blank control*_ represent the relative fluorescence value of the intact enzyme, samples and blank control respectively. The experiment was conducted in triplicates and the IC_50_ values were expressed into mean ± standard deviation.

### 2.7 Screening and identification of potential inhibitors through UF-UPLC/QTOF-MS

#### 2.7.1 UPLC/QTOF-MS identification

The ultra-performance liquid chromatography (UPLC) system was monitored with CDS OpenLAB 2.0 (Agilent Technologies, Santa Clara, United states). The chromatographic separation was carried out using a Sunniest C18 HT (100 mm × 2.1 mm, 2 µm thickness, Waters, Manchester) column. The injection volume was 5 µL with a flow rate kept at 0.2 ml/min while the column temperature was maintained at 30°C. The mobile phases were composed of eluent A (aqueous formic acid, 0.1%) and B (ACN). The elution followed a gradient program of 5%–10% B in 0–5 min, 10% B in 5–15 min, 10%–15% B in 15–30 min, 15%–17% B in 30–35 min, held at 17% B in 35–45 min, finally raised to 70% B from 45–55 min. The components were identified through an Agilent 6530 iFunnel QTOF-MS mass spectrometer equipped with an electrospray ionization (ESI) system. Mass-to-charges and spectra were obtained in negative electrospray ionization mode at a mass range from 50–1,500 m/z. The drying, nebulizing, and collision gas were nitrogen. The drying gas flow rate was 8 L/min with an ESI spray voltage of 3.5 kV. The heated capillary temperature was set to 350°C and nebulizer pressure at 35 psi. The data acquisition and analysis were processed by Mass Hunter Workstation software (version B.08.01.00, Agilent Technology, United States).

#### 2.7.2 Screening for potential inhibitors via bio-affinity ultrafiltration

The bio-affinity screening of potential ligands from the EHNB fraction was performed using a slender modified method by Li and his coworkers (Li et al., 2022). Concisely, 100 µL volume of EHNB sample was incubated with 40 µL COX-2 (8 units), or 100 µL α-Glu (6 units) active enzymes. Afterward, the mix solutions were incubated for 45 min and subsequently transferred to a centrifugal ultrafiltration vial 10 kDa cut-off filter membrane. They were initially centrifuged at 10,000 rpm for 10 min (25°C) to withdraw the extra solution followed by three successive centrifugations with 200 µL phosphate-buffered saline (PBS) to wash away the non-binding compounds. Finally, the fixed ligand candidates were fished out from the complex throughout enzyme denaturation with 200 μL of 70% (v/v) aqueous methanol before centrifugation (10,000 rpm, 10 min). The filtrates from three denaturation processes were gathered and dried on nitrogen blowing before reconstitution in 50 μL of aqueous methanol (50%, v/v) for further analytical analysis.

### 2.8 Molecular docking simulations

The docking simulations were carried out using the AutoDock 4.2 software. Ligand molecules were drawn with ChemBioDraw Ultra 14.0 and converted to 3-Dimensional (3D) structures by ChemBio3D Ultra 12.0. MM2 option was used for energy minimization of the 3D structures, which were further subjected to Gasteiger charges. The crystal receptor structures of α-Glu (PDB id: 3A4A) and COX-2 (PDB id:1CX2) PDB files were downloaded from the protein data bank (www.rcsb.org). Water molecules and initial ligands were removed before adding polar hydrogens and Kolliman charges. Lamarckian genetic algorithm was used for docking calculation of 50 independent genetic algorithm runs within 250 population size and 25 × 10^5^ energy evaluation. The standard docking procedure for a rigid protein and flexible ligands was conducted within a grid map (60 × 60 × 60), centered at the accredited catalytic site. The default settings were used for all other parameters. At the end of docking, the best poses were analyzed for hydrogen bonding, hydrophobic interaction, and binding energy (BE) values using Discovery Studio Visualizer 2021 program (Dassault Systems BIOVIA, San Diego, CA, United States).

### 2.9 Statistical analysis

All experimental tests were performed in triplicate and results were expressed in mean ± standard deviation (SD). The half-maximal inhibitory concentration (IC_50_) values were calculated from the non-linear regression of the dose-response curve analysis function in GraphPad Prism 8.0.1.2 (GraphPad Software, San Diego, California United States). The significance of the results was compared through one-way ANOVA followed by Duncan’s Multiple Range Test (DMRT) on SPSS statistic 22.0.0 (SPSS Inc. Chicago, IL, United States). For all analyses, a difference at *p-*value < 0.05 was regarded as statistically significant.

## 3 Results

### 3.1 Antioxidant activity of *E. humifusa*

Oxidative stress plays key function in the pathogenesis of metabolic disorders, whereas exogenous antioxidants from natural sources enhance the organism’s defense and its redox homeostasis (Abdel-Daim et al., 2019). The antioxidant activities evaluation of the different *E. humifusa* samples showed noticeable potentialities of the *n*-butanol fraction (EHNB) as compared to the others. EHNB exerted the highest scavenging of DPPH and ABTS with an IC_50_ value of 2.45 ± 0.05 μg/ml and 2.04 ± 0.42 μg/ml respectively. Its scavenging abilities were assessed significantly more active than the positive control BHT and Vit C at a *p*-value < 0.05, as illustrated in Table 1. Meanwhile, the FRAP reducing activity of this EHNB fraction was slightly lower than the Vit C and Trolox positive controls having values of 1.45 ± 0.02 mM Fe^2+/^g, 2.04 ± 0.01 mM Fe^2+^/g, and 1.79 ± 0.01 mM Fe^2+/^g, respectively. Similarly, Table 1 also reveals the richness of EHNB fraction in terms of phenolic content (119.45 ± 1.42 mg GAE/g) and flavonoid content (62.28 ± 0.67 mg RE/g) when compared to the others.

**TABLE 1:** The antioxidant activities, total phenolic content (TPC), and total flavonoid content (TFC) of *E. humifusa* samples.

Samples	IC_50_ DPPH (µg/ml)	IC_50_ ABTS (µg/ml)	FRAP (mM Fe^2+/^g)	TPC (mg GAE/g)	TFC (mg RE/g)
EHCE	10.46 ± 1.14^c^	9.10 ± 0.03^c^	0.79 ± 0.01^e^	45.02 ± 0.19^c^	21.71 ± 0.56^c^
EHPE	59.54 ± 0.78^a^	59.67 ± 0.54^a^	0.18 ± 0.01^g^	1.64 ± 0.58^e^	2.06 ± 0.22^e^
EHEA	6.32 ± 0.60^d^	3.78 ± 0.93^e^	1.02 ± 0.01^d^	94.40 ± 0.11^b^	31.45 ± 0.11^b^
EHNB	2.45 ± 0.05^f^	2.04 ± 0.42^f^	1.45 ± 0.02^c^	119.45 ± 1.42^a^	62.28 ± 0.67^a^
EHWA	19.95 ± 0.69^b^	15.87 ± 0.21^b^	0.45 ± 0.02^f^	20.36 ± 0.19^d^	10.06 ± 0.40^d^
BHT	10.82 ± 0.37^c^	Nt	Nt	Nt	Nt
Vit C	3.35 ± 0.12^f^	4.18 ± 0.06^e^	2.04 ± 0.01^a^	Nt	Nt
Trolox	Nt	6.15 ± 0.98^d^	1.79 ± 0.01^b^	Nt	Nt

Note: The values are presented as the mean ± SD, of three replicates; the different superscript letters (a-g) in the same column represent the statistical difference using one way ANOVA DMRT, at *p*-value < 0.05 and Nt means not tested.

### 3.2 α*-*Glu inhibition activity of *E. humifusa*

**FIGURE 1:**
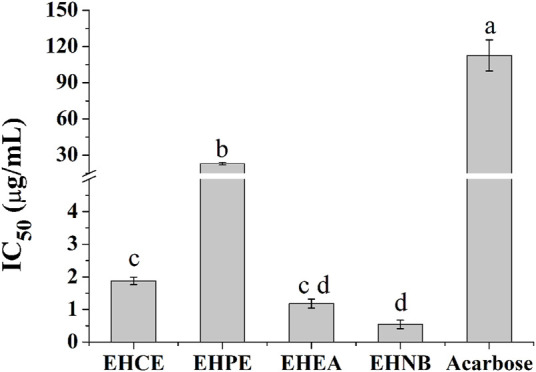
α-Glu inhibitory effects of *E. humifusa* crude extract (EHCE) and the different fractions. Petroleum ether (EHPE), ethyl acetate (EHEA) and *n-*butanol (EHNB). The label letters (a–d) represent the significant difference at *p*-value < 0.05 by ANOVA DMRT.

The hypoglycemic activities of *E. humifusa* extract and its fractions were assessed through *in vitro* α-Glu inhibition assay. The α-Glu enzyme has been reported to play a key role in the catalysis of carbohydrate digestion, therefore, its inhibition leads to a diminution of postprandial glucose absorption. This present study was initially designed to determine the most effective hypoglycemic fraction among the different types of *E. humifusa* samples. The result in Figure 1 exerted that all samples possessed great inhibition of α-Glu in comparison with the well-known antidiabetic drug acarbose. EHNB, possessing a low IC_50_ value of 0.54 ± 0.12 μg/ml, was determined significantly more potent than acarbose (IC_50_: 112.53 ± 12.87 μg/ml), followed by the ethyl acetate fraction (EHEA) and the crude extract (EHCE).

### 3.3 COX-2 inhibition activity of *E. humifusa*

**FIGURE 2:**
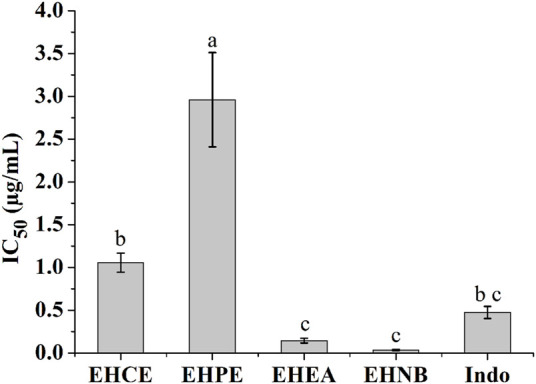
COX-2 inhibitory effects of the *E. humifusa* crude extract (EHCE) and its different fractions. Petroleum ether (EHPE), ethyl-acetate (EHEA) and *n-*butanol (EHNB). The label letters (a−c) represent the significant difference at *p*-value < 0.05 by ANOVA DMRT.

COX-2 enzyme has been recognized to possess a pivotal role in mediating inflammation response by converting the arachidonic acid to prostaglandin G2 and H2. It was discovered that EHNB and EHEA had equipotent COX-2 inhibition activity together with the non-steroidal anti-inflammatory drug (NSAID) indomethacin showing IC_50_ values of 0.03 ± 0.00 μg/ml, 0.14 ± 0.02 μg/ml and 0.47 ± 0.07 μg/ml correspondingly (Figure 2).

As a whole, the *n*-butanol fraction (EHNB) exerted the best activities both as hypoglycemic and anti-inflammatory which were in line with the antioxidant results. Hence, the bioactive components of this potent fraction were further screened out *via* the high throughput bio-affinity ultrafiltration combined with UPLC/QTOF-MS targeting α-Glu and COX-2 enzymes.

### 3.4 Ultrafiltration UPLC/QTOF-MS analysis

#### 3.4.1 Characterization and identification of chemical constituents of EHNB

**FIGURE 3:**
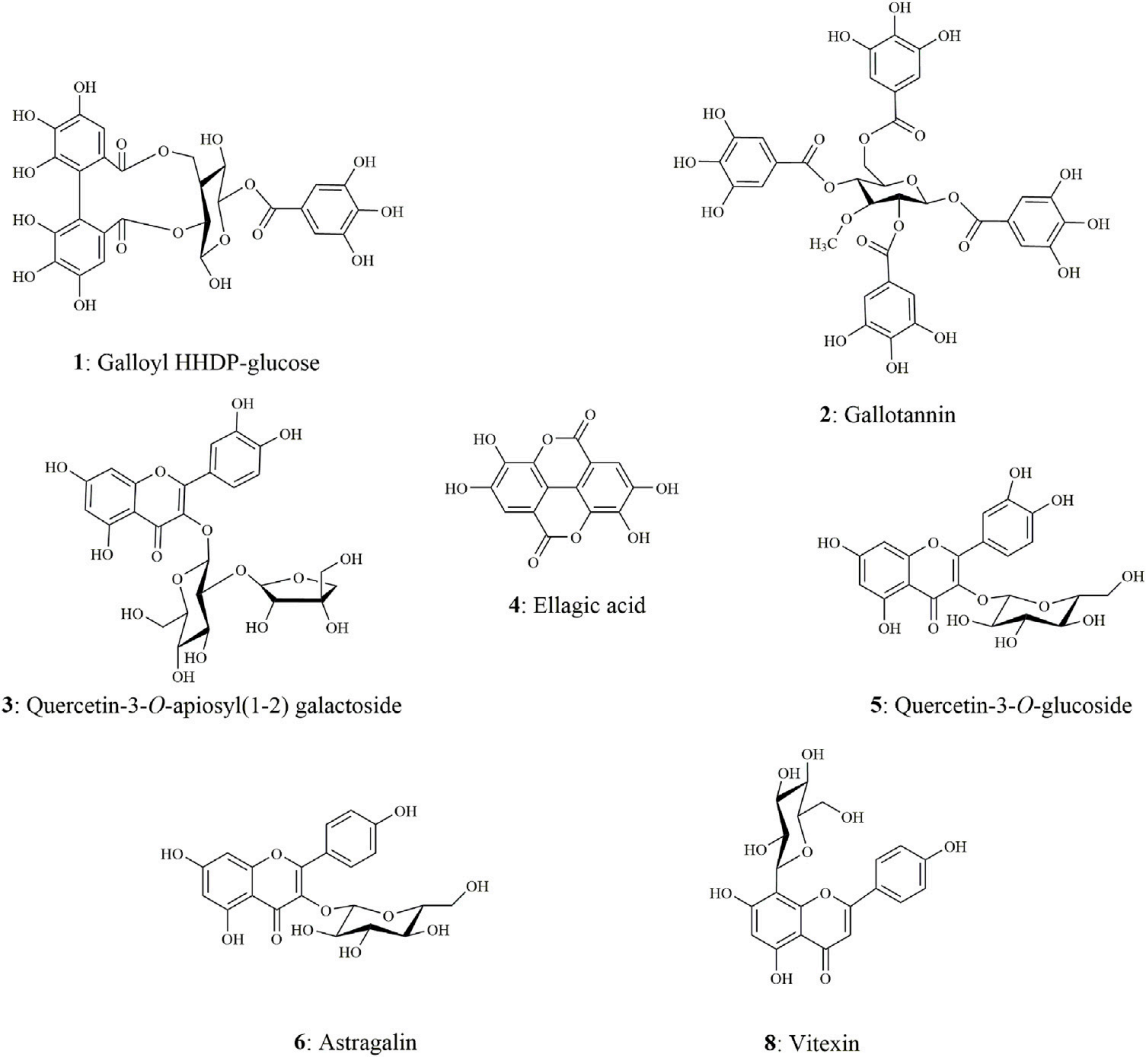
Chemical structures of the seven identified compounds from EHNB.

The chemical components characterization of this potent fraction EHNB was determined by a high-resolution UPLC/QTOF-MS instrument. The tentative identification of all components was performed based on the peak retention time, the parent ions *m/z* with their respective fragmentation cascade as well as the mass error between the observed and theoretical mass. Among the 8 obtained peaks, 7 compounds were identified including tannins, phenolic acid and flavonoid glycosides. The features and characteristics of each identified compounds are displayed in Table 2 and their respective structures illustrated in Figure 3. Two tannins compounds (1, 2) showing precursor ions mass of *m/z* 633.0766 [M-H]^-^ and 800.0822 [M-H]^-^ in the negative mode were respectively assigned as, galloyl HHDP-glucose and gallotanin (Anand et al., 2021). Compound 4**,** with a precursor ion mass of m/z 300.9990 [M-H]^−^, consists of phenolic acid and was tentatively identified as ellagic acid (Camargo et al., 2014). The compounds 3, 5, 6 and 8 belong to flavonoid glycosides, which were respectively deprotonated at *m/z* 595.1299 [M-H]^-^, *m/z* 463.0885 [M-H]^-^, *m/z* 447.0927 [M-H]^-^, and *m/z* 431.0967 [M-H]^-^. According to their features, they were ascribed as quercetin-3-*O*-apiosyl-(1–2) galactoside, quercetin 3-*O*-glucoside, astragalin, and vitexin (Chen et al., 2019; Lu et al., 2022; Rakotondrabe et al., 2022).

**TABLE 2:** The identification of the potential ligands from EHNB and their specific binding values with molecular docking analysis.

Peak No	RT (min)	Observed *m/z*[M-H]^-^	Theoretical *m/z* (Δ ppm)	Characteristic fragments (abundance %)	Tentative identification	α-Glu			COX-2		
						SB	BE (kcal/mol)	IC_50_ (µM)	SB	BE (kcal/mol)	IC_50_ (µM)
1	23.035	633.0766	633.0806 (−6.31)	465.0017 (18.08), 300.9412 (100), 275.0033 (23.96), 168.9701 (64.5)	Galloyl HHDP-glucose	—	—	-—	—	-—	—
2	28.410	801.0822	801.0856 (−4.24)	632.9739 (100), 464.9822 (18.08), 300.9429 (3.17), 169.5332 (2.54)	Gallotanin	-—	-—	—	—-	—	—
3	30.523	595.1299	595.1305 (−1.00)	300.9827 (13.51), 270.9843 (2.61), 178.9503 (1.50), 150.9559 (3.1)	Quercetin-3-*O*-apiosyl-(1–2) galactoside	—	—-	—	—-	—	-—
4	32.988	300.9981	300.9990 (−2.99)	284.2479 (1.25), 256.9575 (3.55), 228.9680 (3.58)	Ellagic acid	1.28	−7.67	Nt	1.05	−6.65	Nt
5	34.423	463.0885	463.0882 (0.65)	300.9767 (69.05), 270.9786. (4.65), 178.9593 (5.97), 150.9683 (19.26)	Quercetin 3-*O*-glucoside	-—	-—	—	—	-—	—
6	34.815	447.0929	447.0933 (−1.34)	284.9838 (100), 255.1797 (1.6), 226.9957 (15.12), 151.0157 (3.54)	Astragalin (Kaempferol -3-*O*-glucoside)	1.32	−8.61	42.47 ± 4.13	1.36	−8.94	49.05 ± 1.49
7	41.285	279.0373		127.9924 (11.3), 96.9934 (100)	Unknown	-—	—-	—-	—	-—	—-
8	44.256	431.0967	431.0984 (3.94)	311.0053 (1.73), 268.9930 (34.89)	Vitexin (Apigenin 8-*C*-glucoside)	1.26	−8.65	36.38 ± 3.06	1.32	−9.19	27.91 ± 1.74

**FIGURE 4:**
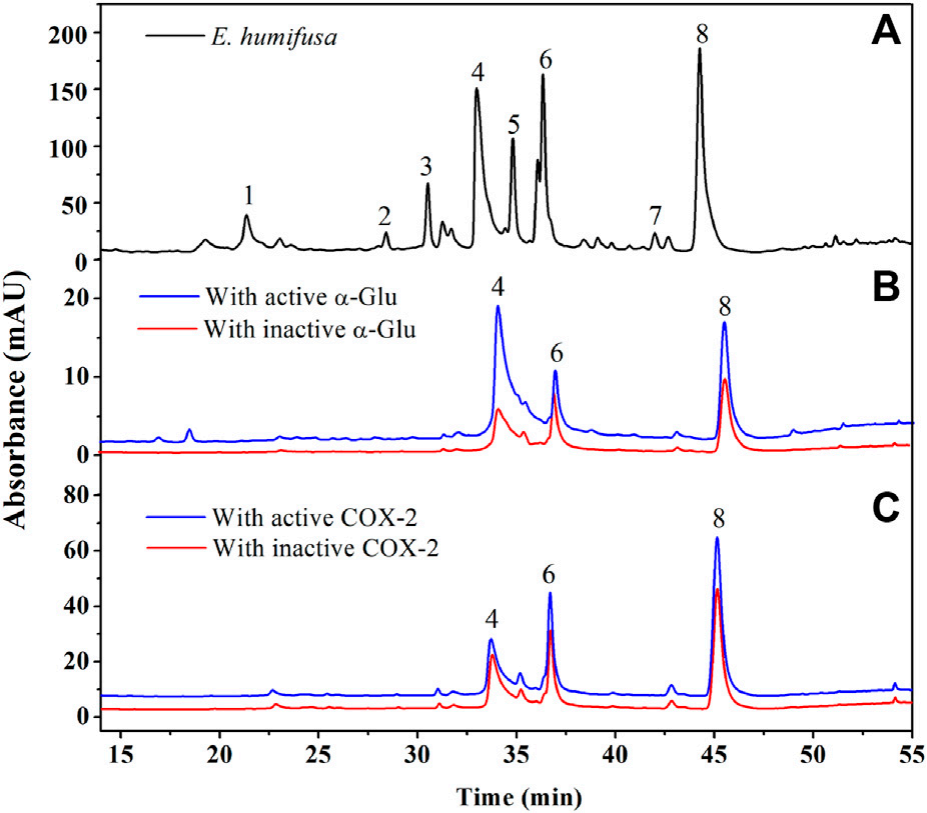
UPLC-UV chromatograms comparison of the EHNB sample (black) EHNB incubated with active enzyme (blue), and inactive enzyme (red) obtained at 360 nm. **(A)** Represents the chromatogram obtained from EHNB sample, **(B)** from α-Glu target and **(C)** from COX-2 target.

Peak No. And retention time (RT) correspond to the UPLC-UV chromatogram of Figure 4; SB represents the specific binding; BE represents the binding energy; Nt means not determined, and - stands for nothing.

#### 3.4.2 Discovery of potential α-Glu and COX-2 ligand candidates

Within medicinal plants, one or more bioactive components are attributed to be responsible for their biological activities. In this study, the high throughput ligand screening bio-affinity ultrafiltration UPLC/QTOF-MS targeting α-Glu and COX-2 has been utilized to fish out these key hypoglycemic and anti-inflammatory chemicals. This method has been testified efficient and reliable by several researchers to depict potential ligands from a complex mixture. Based on the comparison of peak areas of the chromatograms obtained from the sample incubated with the active enzyme and that of the denatured group, the relative binding of each candidate was assessed via the specific binding value calculated from the equation:

$$\text{Specific binding }\left(\text{SB}\right)={\text{A}}_{active}/{\text{A}}_{inactive}\;$$

With A_*active*_ and A_*inactive*_ corresponding to the area of the peaks within the chromatogram of those incubated with active and inactive enzymes. From our experiment, it was discovered that three compounds showed an affinity with the two targets, notably ellagic acid, astragalin, and vitexin (Figure 4). Astragalin showed the highest specific binding (SB) value of 1.32 with α-Glu followed by ellagic acid (SB: 1.28) and vitexin (SB: 1.26). However, vitexin took the second rank when targeting the COX-2 enzyme with the respective SB values of 1.36 (astragalin), 1.32 (vitexin), and 1.05 (ellagic acid). These rates suggested that these three compounds could be speculated as ligand candidates for our targets. Additionally, their interactions with the target’s active site were simulated *in silico* to in-depth their affinity and appended to the *in vitro* validation activities assessment.

### 3.5 Molecular docking analysis

**FIGURE 5:**
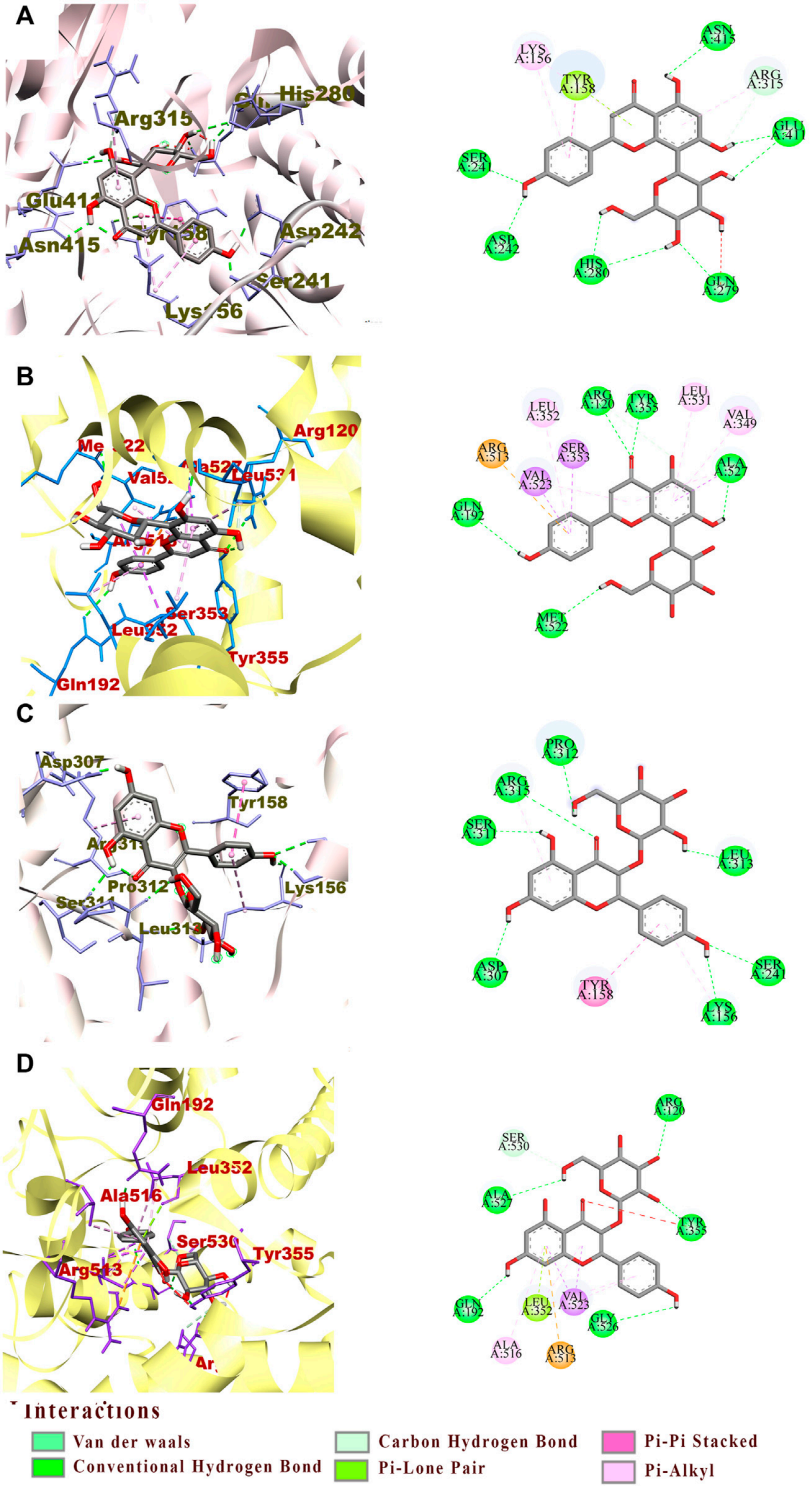
The interactions between active pocket of α-Glu (grey) or COX-2 (yellow) with vitexin **(A,B)** and astragalin **(C,D)** by molecular docking analysis. The key interacting amino acid residues are: Serine (Ser), Glutamine (Gln), Arginine (Arg), Glutamic acid (Glu), Tyrosine (Tyr), Lysine (Lys), Aspartic acid (Asp), Proline (Pro), Leucine (Leu), Valine (Val), and Glycine (Gly).

For a better understanding of the binding mechanism and interaction of these ligand candidates with the target receptor, *in silico* molecular docking simulations were conducted. Simultaneous docking of the respective positive drugs acarbose and indomethacin into the active site of α-Glu and COX-2 were executed to validate the docking method. The root mean square deviation value (RMSD) obtained from the docking validation of the complex acarbose-α-Glu was 1.010 ± 0.265 Å when 0.643 ± 0.038 Å for indomethacin-COX-2. The ligand receptor complex binding stability was assigned based on the BE values of the best docking pose; from which the lower the value, the stronger the interaction. Toward α-Glu, the highest stability was found with vitexin exhibiting a BE value of −8.65 kcal/mol. Its interaction was explained by the occurrence of nine hydrogen bonds toward the key residues Ser241, Gln279, Arg315, Glu411, His280, Asn415, and Asp242 (Figure 5A). In addition, hydrophobic binding was determined with the pocket constituted by Tyr158, Arg315, and Lys156. The astragalin exerted BE value of −8.61 kcal/mol formed of eight hydrogen bonds with Ser241, Arg315, Asp307, Ser311, Lys156, Pro312, and Leu313 key residues. The astragalin-α-Glu complex was stabilized by hydrophobic interactions between the ligand and the pocket established with Tyr158 and Lys156 (Figure 5C). The weakest complex stability was identified with ellagic acid, showing a BE value of −7.67 kcal/mol. Toward COX-2, vitexin remained the greatest bound ligand with a BE value of −9.19 kcal/mol. The interactions with the catalytic site were composed of six hydrogen bonds to the key residues Arg120, Tyr355, Ser530, Ala527, Met522, and Gln192 (Figure 5B). Electrostatic and hydrophobic interactivity between vitexin and the pocket were also present and formed of Arg513, Val349, Ala527, Leu531, Val523, and Leu352. However, the astragalin-COX-2 BE value was −8.94 kcal/mol, made by seven hydrogen bonds with the residues Arg120, Tyr355, Ala527, Ser530, Gln192, and Gly526 (Figure 5D). The same electrostatic interactivity with Arg513 was identified and supplemented by hydrophobic bonds with Ala516, Val523, and Leu352. Likewise, the lowest stability was found on the ellagic acid-COX-2 complex with a BE value of −6.45 kcal/mol. Due to the low relative binding values along with the poor stabilities established between the ellagic-acid and targets, only vitexin and astragalin were speculated as potential ligands toward α-Glu and COX-2. The *in-vitro* confirmation of their activities was then investigated.

### 3.6 Potential ligands activities validations

**FIGURE 6:**
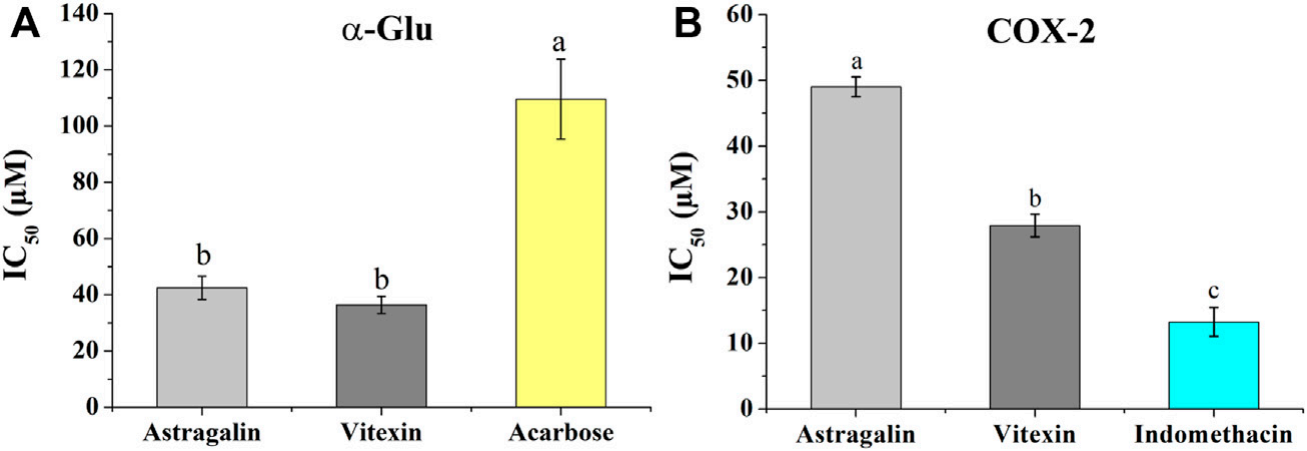
Inhibition effects of astragalin and vitexin compared to the respective positive controls on α-Glu **(A)** and COX-2 **(B)**. The label letters **(a,b,c)** represent the significant difference at *p*-value < 0.05 by ANOVA DMRT.

To validate the hypoglycemic and anti-inflammatory activities of the two screened-out potential ligands, the same colorimetric *in vitro* inhibition assays as for the preliminary *E. humifusa* crude extract and its fractions activities assessment were conducted. As illustrated in Figure 6A, astragalin and vitexin exerted interesting equipotent activities against α-Glu with a noticeable IC_50_ of 42.47 ± 4.13 µM and 36.38 ± 3.06 µM accordingly. They were statistically more active than the positive drug acarbose showing an IC_50_ of 109.54 ± 14.23 µM. On the other hand, the two-screened ligands showed moderate COX-2 inhibition capacity in comparison to the positive NSAID indomethacin as displayed in Figure 6B. Vitexin exhibited a greater half-maximal inhibitory concentration of 27 0.91 ± 1.74 µM than astragalin 49.05 ± 1.49 µM whereas indomethacin has an IC_50_ of 13.24 ± 2.20 µM.

## 4 Discussion

### 4.1 Antidiabetic and anti-inflammatory activities of *E. humifusa*

DM is a ubiquitous health disorder resulting from insulin deficiency or malfunction, which leads to a high rate of glucose in the blood (Kazeem et al., 2021). Research revealed that a high level of blood glucose enhances oxidative stress at the cell level, which boosts the pro-inflammatory cytokines activities, such as interleukins, tumor necrosis factors as well as mediators like nitric oxide. The aforementioned cytokines induce the expression of COX-2 and increase prostaglandin synthesis, implying that diabetes plays a crucial role in the chronic inflammatory state (Burgos-Morón et al., 2019; Kaur and Singh, 2022). The side effect and toxicity of most actual existent drugs urges researchers to seek for safe and effective nature-based agents to manage this prevalence and debilitating complication of DM. Reducing postprandial hyperglycemia is one of the relevant therapeutic approaches for DM, which can be achieved by inhibiting digestive enzymes such as α-Glu. In the same way, blocking the COX-2 enzyme function is among the principal strategy to regulate inflammation (Karim et al., 2019). Although *E. humifusa* has been used to cure a variety of health disorders including hyperglycemia and inflammation-based diseases, the scientific reports describing the key bioactive compounds in this species remain elusive. Therefore, in this present study, we have determined primarily the biological potential of *E. humifusa* crude extract and its fractions followed by the characterization and screening of the bioactive ligands through bio-affinity UPLC/QTOF- MS.

The preliminary *in vitro* evaluation of all *E. humifusa* samples showed good antidiabetic activity, which is in line to the investigation of Kang and his coworkers, demonstrating the noticeable capacity of *E. humifusa* methanol extract to inhibit α-Glu (Kang et al., 2012). Among these samples, the *n-*butanol fraction (EHNB) was discovered to be the most active part with an IC_50_ value of 0.54 ± 0.12 μg/ml and significantly more potent than the common hypoglycemic acarbose drug. Similarly, the anti-inflammatory activity of EHNB was assessed the best, even when compared to the NSAID indomethacin. Therefore, our finding enhances prior discoveries demonstrating the anti-inflammatory of this species through inhibition of soluble epoxide hydrolase (SEH), lipopolysaccharide (LPS)-induced nitric oxide and the tumor necrosis factors (TNF) productions (Luyen et al., 2014). Interestingly, the antioxidant evaluations via DPPH, ABTS and FRAP assays showed the highest potentialities of the EHNB fraction. The close correlations between these EHNB favorable pharmacological effects are consistent with previous findings describing the relevance of Euphorbiaceae-based antioxidants in controlling the initiation or propagation of other chronic diseases (Majid et al., 2015; Mustafa et al., 2021). Furthermore, the richness of this EHNB fraction with phenolic and flavonoids (Table 1) may support the multiple drug activities of this plant due to the abilities of plant polyphenols to modulate various enzymes and immune cells in human, in spite their antioxidant potentials (Sekhon-Loodu and Rupasinghe, 2019; Sun et al., 2020).

### 4.2 Potential inhibitors in EHNB and their docking poses

**FIGURE 7:**
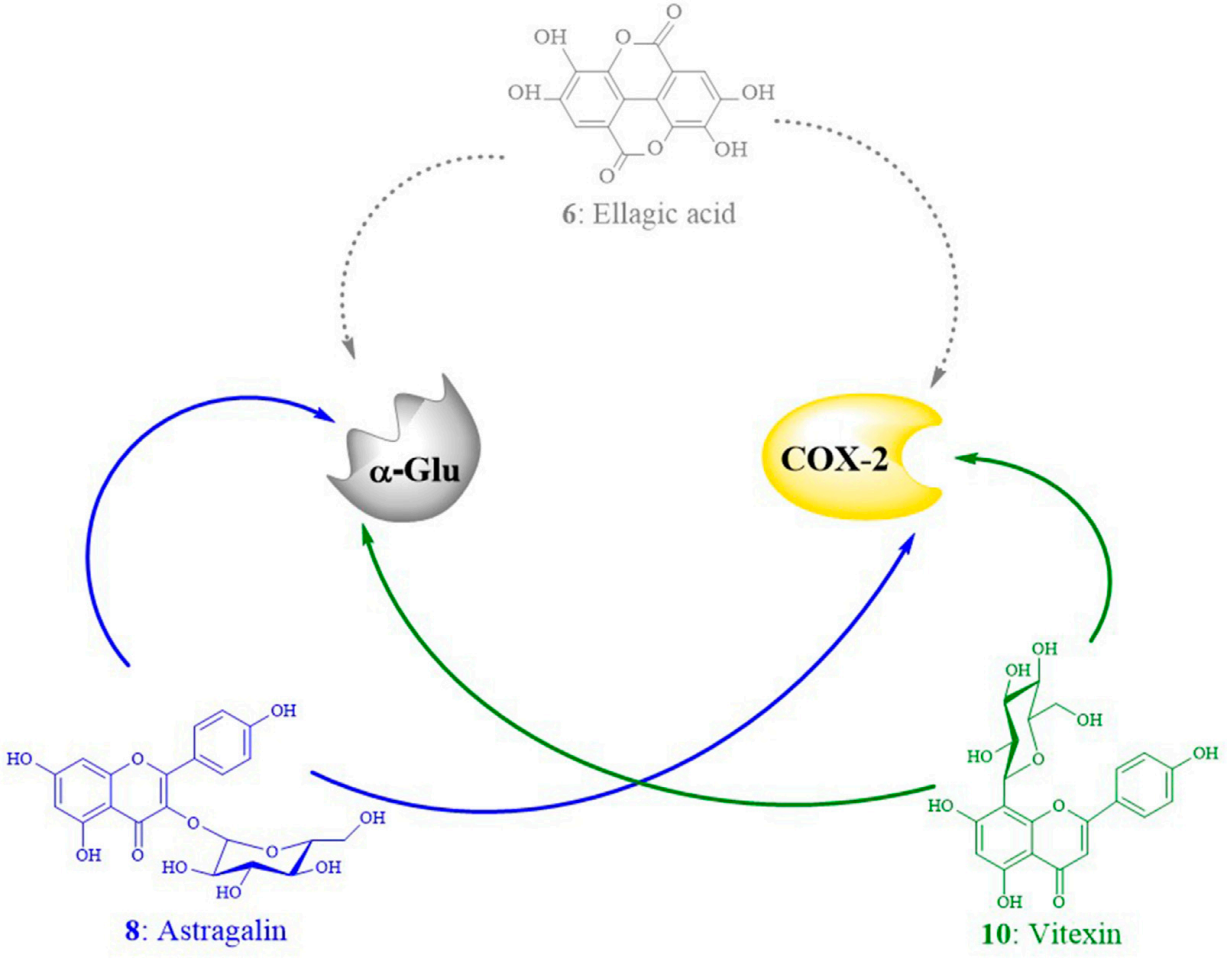
Interaction networks diagram of the ligand candidates from *E. humifusa* toward the enzyme targets α-Glu and COX-2. The dotted arrows reflect weak ligand affinities toward the targets active site, whereas the plain ones represent strong affinities.

The chemical characterization of EHNB allowed us to identify seven phytoconstituents of which one phenolic acid named ellagic acid and two flavonoid glycosides astragalin and vitexin were fished out as ligand candidates through bio-affinity ultrafiltration UPLC/QTOF-MS. Astragalin and vitexin were selected as potential inhibitors due to their relative binding strength values together with the *in silico* BE values, whose activities were further verified (Xu et al., 2022; Ye et al., 2022; Zhang et al., 2022). As illustrated in Table 2, those two flavonoid glycosides showed better SB values towards the enzyme targets α-Glu and COX-2. This binding strength diversity was speculated to be caused by the competitive interaction of the ligand candidates with the target enzymes. Additionally, these two ligands were docked well in the catalytic site of the targets with a BE values lower than the respective drugs acarbose and indomethacin. The network interaction diagram (Figure 7) highlights the discovery of the potential ligands vitexin and astragalin as well as their double acting actions. Both vitexin and astragalin interacted with the recognized key amino acid residues of α-Glu catalytic pocket composed of Arg315, Glu411, His280, Asp242, Asp307, Ser311, Pro312, Leu313, Tyr158, and Lys156 (Cai et al., 2020; Nokhala et al., 2020). The lower BE value of vitexin (−8.65 kcal/mol) was supported by the presence of one extra H-bond with GLU 411 which was absent for astragalin (−8.61 kcal/mol). It is emphasized that α-Glu complexes formed with those potent ligands were far more stable than those made with the antidiabetic drug acarbose, which had just a BE value of −5.25 kcal/mol. In the meantime, vitexin was found more stable within the COX-2 catalytic site than astragalin due to H-bonds number differences and hydrophobic interactions. Nonetheless, both of them revealed better BE value than the NSAID indomethacin which were ranked as follows vitexin (−9.19 kcal/mol)> astragalin (−8.94 kcal/mol)> indomethacin (−6.64 kcal/mol). The interactions of those potential ligands with the reported important key residues Arg120, Arg513, Tyr355, and Val523 inside the active pocket are consistent with the intercalation of selective COX-2 inhibitors docking (Baek et al., 2021).

### 4.3 Vitexin and astragalin activities validations

The antidiabetic *in vitro* validation results synthesized in Figure 6 and Table 2 demonstrated that vitexin had better activity than astragalin with an IC_50_ of 36.38 ± 3.06 µM and 42.47 ± 4.13 µM, respectively. Both candidates exerted a significant inhibition potentiality than the well-known hypoglycemic drug acarbose possessing an IC_50_ of 109.54 ± 14.23 µM. Recent studies have highlighted the relevance of vitexin in glucose homeostasis. It helps in the protection of pancreatic tissues from damage, enhances the uptake of glucose, and modulates the catalytic function of the two main digestive enzymes α-amylase and α-Glu (He et al., 2016; Seyedan et al., 2019; Abdulai et al., 2021). In the same way, previous investigations have highlighted the hypoglycemic effectivities of astragalin through inhibition of key enzymes like α-Glu, α-amylase, and tyrosine phosphatase (PTP 1B) (Cen et al., 2017; Lima et al., 2018). The *in vitro* anti-inflammatory validation exhibited good COX-2 inhibition activity of vitexin with an IC_50_ value of 27.91 ± 1.74 µM. This result was in support of prior studies, which demonstrated the anti-inflammatory activity of vitexin via downregulation of pro-inflammatory cytokines releases, as well as reducing the actions of inflammatory mediators like COX-1 and COX-2 (Babaei et al., 2020; Abdulai et al., 2021; Peng et al., 2021). Likewise, the moderate COX-2 inhibition of astragalin (IC_50:_ 49.05 ± 1.49 µM) contributed to the explanation of its multifaceted activity to mitigate inflammation. Therefore, besides astragalin’s capacities to block mitogen-activated protein kinases (MAPK) signaling pathways and to inhibit the production of inflammatory mediators like prostaglandin E2 (PGE2) (Zhang et al., 2017; Alblihed, 2020), here it is supported to regulate COX-2 function.

Taken together, the finding from this study helps to understand the principal bioactive compounds playing pivotal roles in the multifarious empirical uses of *E. humifusa.* The two screened-out active ligands (vitexin and astragalin) were confirmed to exert potential antidiabetic and anti-inflammatory activities. Therefore, the development of natural therapeutic drug candidates for diabetes mellitus and its associated complications based on those two flavonoids could be promising.

## 5 Conclusion

DM and its associated complications have devastated human beings in the last 2 decades. So far, *E. humifusa* has been reported to possess multiple virtues including its antidiabetic and anti-inflammatory activities. The present investigation was designed to screen out the potential double-acting bioactive compounds and their mechanism of action toward hyperglycemia and inflammation. Integrated method comprising the high throughput bio-affinity ultrafiltration combined with UPLC/QTOF-MS targeting α-Glu and COX-2 enzymes, *in vitro* assays, and *in silico* study were adopted throughout the research. The *n*-butanol (EHNB) fraction was revealed to be the best active fraction, from which two flavonoid glycosides vitexin and astragalin were fished out as potential ligands. The *in silico* simulation showed their great stability within the active site of the two target enzymes. Meanwhile, the validation activity assays confirmed their capacities to inhibit concomitantly α-Glu and COX-2. To sum up, this present investigation offered additional scientific support for the antidiabetic and anti-inflammatory use of *E. humifusa* and facilitated eliciting the modes of action of its potential active compounds.

## Data Availability

The raw data supporting the conclusion of this article will be made available by the authors, without undue reservation.
